# Dr. Bouillaud’s Election to the Chamber of Deputies

**Published:** 1842-11-26

**Authors:** 


Dr. Bouillaud, the distinguished professor and physician of La Charité, at Paris, whose election to the Chamber of Deputies we noticed some time since, lost his seat by some informality in the proceedings. He has been lately reëlected for Angoulême by a majority of forty-six votes.

